# Clinical, genomics and networking analyses of a high-altitude native American Ecuadorian patient with congenital insensitivity to pain with anhidrosis: a case report

**DOI:** 10.1186/s12920-020-00764-3

**Published:** 2020-08-17

**Authors:** Andrés López-Cortés, Ana Karina Zambrano, Patricia Guevara-Ramírez, Byron Albuja Echeverría, Santiago Guerrero, Eliana Cabascango, Andy Pérez-Villa, Isaac Armendáriz-Castillo, Jennyfer M. García-Cárdenas, Verónica Yumiceba, Gabriela Pérez-M, Paola E. Leone, César Paz-y-Miño

**Affiliations:** 1 Centro de Investigación Genética y Genómica. Facultad de Ciencias de la Salud Eugenio Espejo, Universidad UTE, Mariscal Sucre Avenue, 170129 Quito, Ecuador; 2 Latin American Network for Implementation and Validation of Clinical Pharmacogenomics Guidelines (RELIVAF-CYTED), Madrid, Spain; 3 Hospital San Luis de Otavalo, Ministerio de Salud Pública, Antonio José de Sucre Avenue, 100201 Otavalo, Ecuador; 4 Sistemas Médicos (SIME), Universidad San Francisco de Quito, Interoceánica Avenue and Chimborazo, 170902 Cumbayá, Ecuador; 5 Ministerio de Salud Pública, 100117 Ibarra, Ecuador

**Keywords:** CIPA, Native American, Ecuadorian, *NTRK1*, Genomics analysis

## Abstract

**Background:**

Congenital insensitivity to pain with anhidrosis (CIPA) is an extremely rare autosomal recessive disorder characterized by insensitivity to pain, inability to sweat and intellectual disability. CIPA is caused by mutations in the neurotrophic tyrosine kinase receptor type 1 gene (*NTRK1*) that encodes the high-affinity receptor of nerve growth factor (NGF).

**Case presentation:**

Here, we present clinical and molecular findings in a 9-year-old girl with CIPA. The high-altitude indigenous Ecuadorian patient presented several health problems such as anhidrosis, bone fractures, self-mutilation, osteochondroma, intellectual disability and Riga-Fede disease. After the mutational analysis of *NTRK1*, the patient showed a clearly autosomal recessive inheritance pattern with the pathogenic mutation rs763758904 (Arg602*) and the second missense mutation rs80356677 (Asp674Tyr). Additionally, the genomic analysis showed 69 pathogenic and/or likely pathogenic variants in 46 genes possibly related to phenotypic heterogeneity, including the rs324420 variant in the *FAAH* gene. The gene ontology enrichment analysis showed 28 mutated genes involved in several biological processes. As a novel contribution, the protein-protein interaction network analysis showed that NTRK1, SPTBN2 and GRM6 interact with several proteins of the pain matrix involved in the response to stimulus and nervous system development.

**Conclusions:**

This is the first study that associates clinical, genomics and networking analyses in a Native American patient with consanguinity background in order to better understand CIPA pathogenesis.

## Background

Congenital insensitivity to pain with anhidrosis (CIPA), also known as hereditary sensory and autonomic neuropathy Type IV (HSAN-IV) (OMIM #256800), is an extremely rare autosomal recessive disorder characterized by axonal atrophy affecting the sensory and autonomic neurons [1, 2]. HSAN-IV is characterized by recurrent episodes of unexplained fever, self-mutilating behavior, anhidrosis, absence of reaction to noxious stimuli, intellectual disability [3], humoral immunodeficiency [4], palmoplantar keratoderma [5, 6], and early onset renal disease [7]. This condition occurs with an incidence of 1 in 125 million newborns [8].

Brain regions with pain perception are complex and have been best described as a pain matrix [9, 10]. According to Foulkes et al (2008), it consists of four phases in which different genes/proteins are involved [10]. Nerves inside the skin have the ability to transmit the sensation of heat, cold and mechanical stimulation; Na^+^ and K^+^ channels drive nerve stimuli; the synaptic transmission occurs in the spinal cord via neurotransmitter receptors and Ca^2+^ channels; lastly, central, peripheral and microglia modulation occurs in brain [10]. Nevertheless, patients with CIPA may present genetic alterations causing functional disruption in one of these pain matrix phases.

Only some hundred of cases of CIPA have been reported worldwide [8, 11]. The first reference to a similar pathology was mentioned by Dearborn in 1932 [12], and it was published in 1963 by Swanson [13]. Tunçbilek et al (2005) determined three clinical representative findings: insensitivity to pain, inability to sweat and intellectual disability [14]. Indo et al (1996) associated CIPA pathogenesis with genetic loss-of-function mutations of the *NTRK1* (neurotrophic receptor tyrosine kinase 1) gene [15]. Grills and Schuijers (1998) postulated that NGF function disruption also causes an altered process of fracture consolidation [16]. Indo et al (2001) determined that CIPA is not only an autosomal recessive disorder, but also a uniparental disomy [17]. Jarade et al (2002) observed ocular manifestations [18]. Many studies of Weier et al (1995), Miura et al (2000), Indo et al (2001), Mardy et al (2001), Bonkowsky et al (2003) and Lin et al (2010) discovered novel mutations and polymorphisms in *NTRK1* causing CIPA [19–23]. Schreiber et al (2005) analyzed insulin-related difficulties [24]. Brandes and Stuth (2006) evaluated anesthetic considerations [25]. Many studies of Tanaka et al (1990), Indo (2002) and Melamed et al (2004) determined that NGF receptor failure causes a deficient development of dorsal root neurons (pain and temperature sensory system) autonomic sympathetic neural system (eccrine sweat glands innervation) [26, 27]. Abdulla et al (2014) observed heterotopic ossification and callus formation following fractures and eventually Charcot’s joint [28]. Franco et al (2016) proposed that mutations of *NTRK1* generate different levels of cell toxicity, which may provide an explanation of the variable intellectual disability observed in CIPA [29]. Altassan et al (2016) identified novel *NTRK1* mutations in CIPA individuals through exome DNA sequencing [1]. Finally, Habib et al (2019) identified a microdeletion and a polymorphism (rs324420) in the *FAAH* gene with high anandamine concentrations and pain sensitivity [30].

*NTRK1*, also known as *TRKA*, encodes the neurotrophic tyrosine kinase-1 receptor, which is autophosphorylated activating various intracellular signaling transduction such as cell survival, growth and differentiation [1, 26, 31]. Additionally, the pain insensitivity is caused by the absence of the NGF-dependent primary afferents, and anhidrosis is explained by the lack of the sympathetic postganglionic neurons [1, 32]. According to the Human Gene Mutation Database and the ClinVar, *NTRK1* has ~ 79 alterations among single nucleotide polymorphisms (SNPs), insertions and deletions, inherited in an autosomal recessive pattern [33, 34]. Indo et al (1996) has reported for the first time *NTRK1* mutations associated with CIPA in an Ecuadorian family [15]. However, this is the first time that clinical, genomics, protein-protein interaction (PPi) networking and gene ontology (GO) enrichment analyses were performed in a high-altitude Native American (indigenous) patient with CIPA disease and family consanguinity background.

## Case presentation

The Human Research Ethics Committee from Universidad San Francisco de Quito (No. 2018-127E) approved all experimental protocols. The methods were carried out in accordance with the relevant guidelines and regulations. Lastly, written informed consent to participate was obtained from all of the participants in this study. In case of CIPA patient, a written informed consent to participate was obtained from their parents.

The case of a 9-year-old girl who was born in the community of Piaba (2418 m above sea level, MASL), in Cotacachi, located in the north of Ecuador, is presented. She is diagnosed with CIPA, which begins to be suspected after 72 h of life, where she developed fever of unknown origin; consequently, she was admitted to the hospital, she stayed there for 26 days and she was discharged without specific diagnosis. After 1 month of age, she presented recurring episodes of fever.

**Fig. 1:**
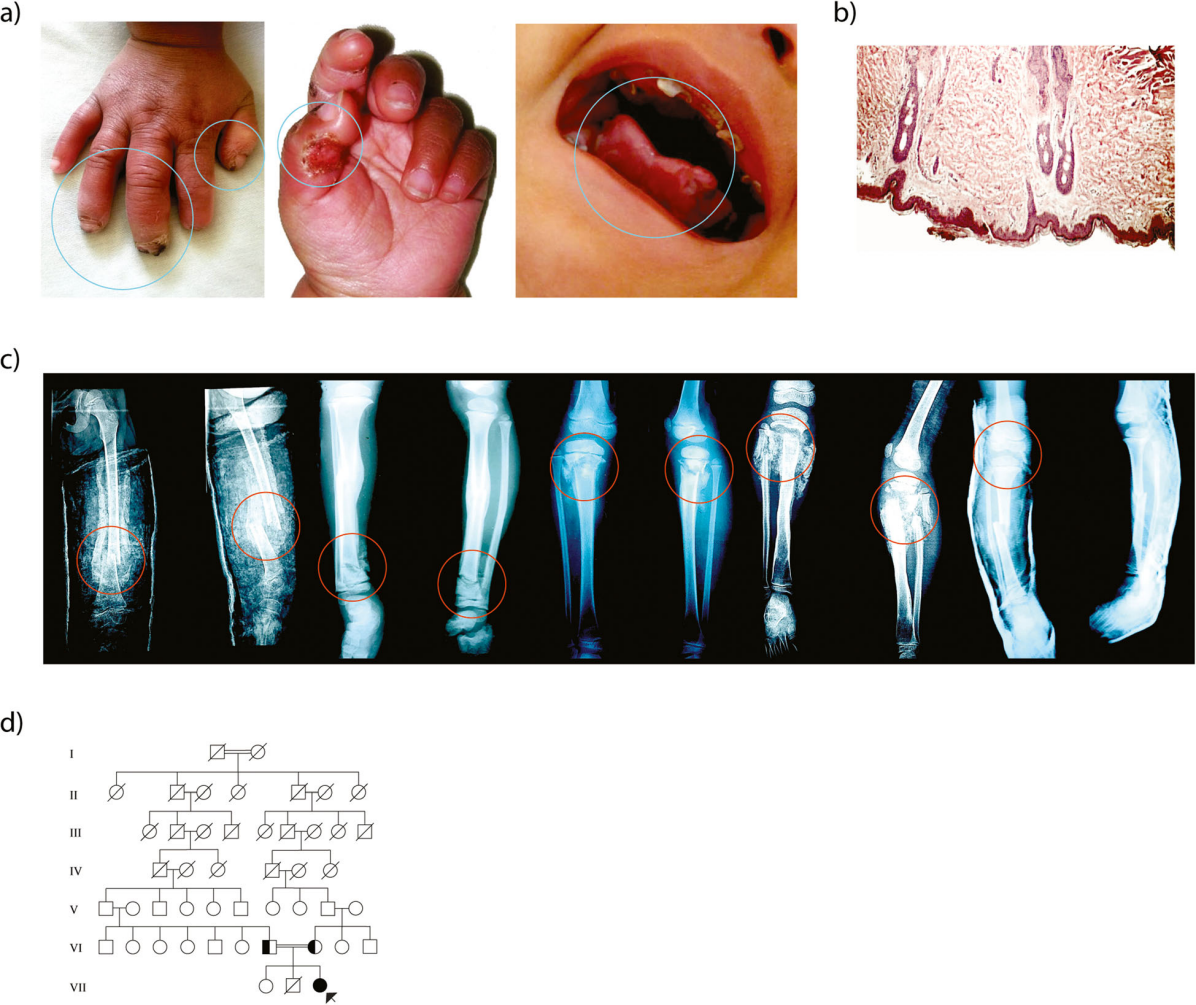
Clinical features of CIPA patient. **a** Self-mutilation. **b** Skin biopsy shows a thin epidermis with hyperkeratosis, there are few sebaceous glands and no nerve terminals are observed. **c** Several fractures in tibia and femur. **d** Family genealogical tree

When she was 4 months old, she was diagnosed with pneumonia; while she stayed at the hospital, her neurodevelopment was examined by means of the Denver test which provided the following result: unusual for her age. The neurological examination showed generalized hypotonia, active symmetrical movements, incomplete cephalic support, absence of pain sensitivity during peripheral line placement, normal deep tendon reflex, and absence of corneal reflex. Additionally, it was observed that there was a 1-cm dermal ulcer, with regular edges on the proximal and distal phalange of the first finger of the right hand, and lesions in healing process on the second finger (Fig. 1a); consequently, with general anesthesia, a skin biopsy of the sternal region was taken to analyze presence or absence of nerve terminals by immunohistochemical staining for S100 protein (Fig. 1b). The negative results showed superficial and deep dermis with some cutaneous appendices constituted by hair follicles and sweat glands that in multiple cuts have no innervation zones, which can be corroborated since the mother presents absence of perspiration.

When she was 16 months old, she was diagnosed with Riga-Fede disease because she presented ulcerative plaques in the oral mucosa and tongue deformities (Fig. 1a). Furthermore, due to previous ulcers, the patient developed osteomyelitis on the distal phalanges of first and second fingers of the left hand (positive culture for *Staphylococcus aureus* sensitive to cephalexin). At 2.5 years old, and later at 3 years and 2 months old she presented bilateral corneal ulcers of traumatic origin. At 6 years and 4 months old she suffered a tibia fracture caused by falling (Fig. 1c). At 6 years and 7 months she presented a distal tibial fracture without a determined cause. During the consolidation process a mass was found in the fracture area; a biopsy was carried out and osteochondroma was diagnosed. At 8 years and 1 month she broke her left femur because of a fall. Finally, at 8 years and 5 months old she suffered a subtrochanteric fracture of the right femur, requiring surgery (Fig. 1c). As for the family background, a sister of the patient passed away at 18 months old after developing fever of unknown origin. Figure 1d details the genealogical tree, and consanguinity between relatives of both parents. In addition, the patient presented a normal karyotype 46,XX (Figure S1).

Regarding methodology, peripheral blood samples were extracted from CIPA patient and her parents using FTA buffer (GE Healthcare, UK). Genomic DNA was extracted from whole blood using the PureLink Genomic DNA Kit (Invitrogen, Carlsbad, CA), and purified using Amicon Ultra centrifugal filters (Merck, Darmstadt, Germany). Genomic DNA presented a concentration of 45 ng/μL (mother), 27 ng/μL (father) and 36 ng/μL (patient), using a Qubit 4 (Thermo Scientific, Waltham, MA).

Genotyping was performed through PCR reaction of 54 SNPs located into 14 regions of *NTRK1* and Sanger sequencing analysis. Table S1 details features of primers and all 54 variants analyzed. Figures S2a and S2b detail the PCR and the sequencing analysis protocols for all genetic variants, respectively. After carrying out the sequencing analysis of the *NTRK1* gene, the patient was observed to have the homozygous mutant genotype T/T of the missense mutation rs80356677 (Asp674Tyr). At the nucleotide level there is a change of the guanine (G) to thymine (T) in DNA. Regarding parents, both have the heterozygous genotype G/T of the mutation rs80356677 (Figure S2c).

Furthermore, a trio analysis of genomic DNA of mother, father and patient was done by using the TruSight One (TSO) Next-Generation Sequencing (NGS) Panel (Illumina, Inc. San Diego, CA, USA), which includes 125,395 probes targeting a 12-Mb region spanning ~ 62,000 target exons of 4811 genes, and sequenced on the Illumina MiSeq platform. Raw sequence reads were processed and aligned against the human NCBI GRCh37 hg19 reference genome assembly using the BWA software. The 80-mer probes target libraries with ~ 500 bp mean fragment sizes and ~ 300 bp insert sizes, enriching a broad footprint of 350–650 based centered symmetrically around the midpoint of the probe. Therefore, in addition to covering the main exon regions, the panels cover exon-flanking regions, which can provide important biological information such as splice sites or regulatory regions. The TSO coverage was ≥20x on 95% of the target regions in the panel, and the TSO full gene list is detailed in the Table S2.

**Fig. 2:**
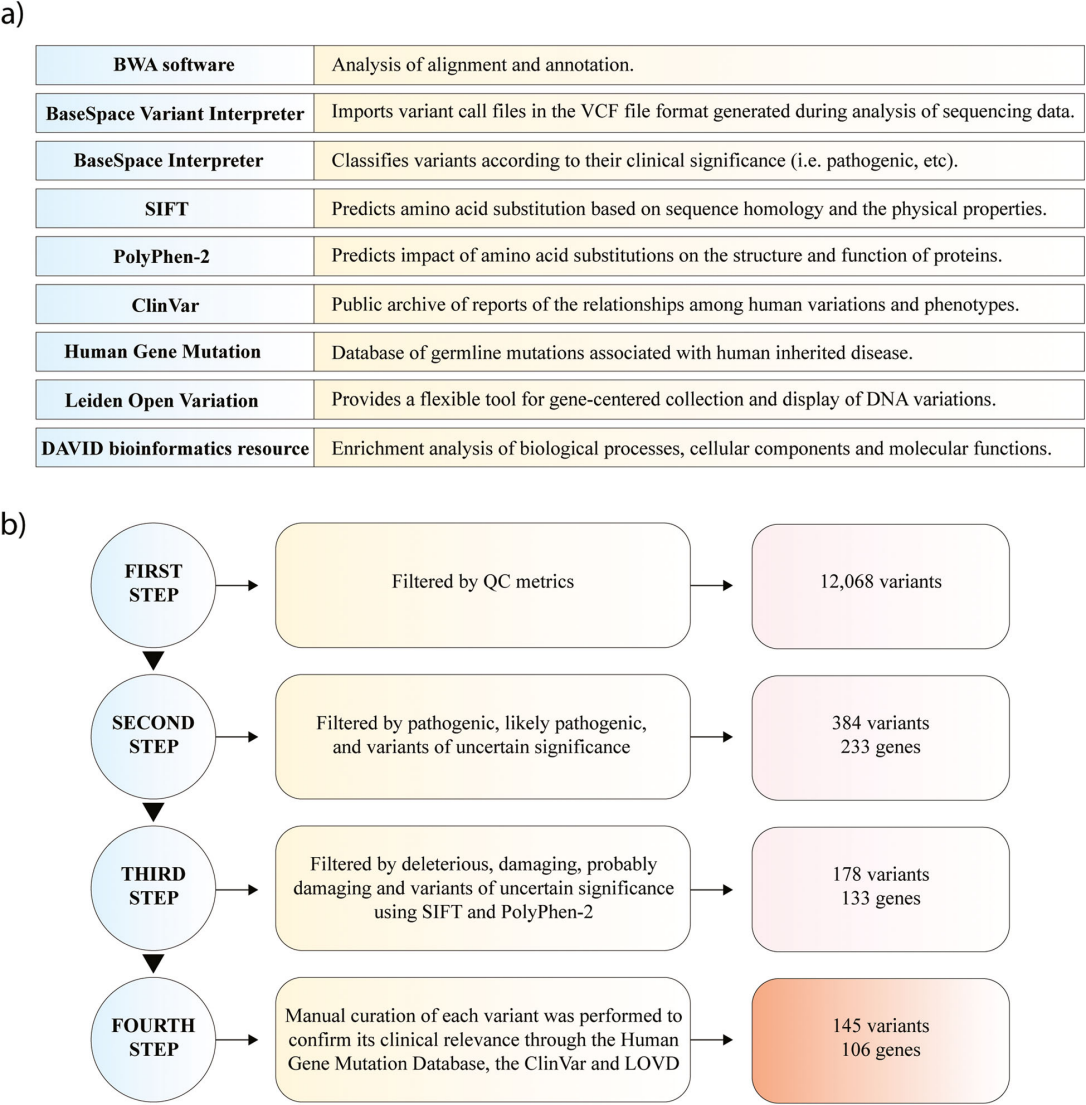
Next-generation sequencing analysis. **a** Functions of software and databases used for NGS analysis. **b** Pipeline of genomic variant analysis

To analyze data generated from targeted sequencing, the BaseSpace Variant Interpreter software (Illumina), the BaseSpace Interpreter (Illumina), Sorting Intolerant From Tolerant (SIFT) (http://sift.bii.a-star.edu.sg/) [35], Polymorphism Phenotyping v2 (PolyPhen-2) (http://genetics.bwh.harvard.edu/pph2/) [36], ClinVar (https://www.ncbi.nlm.nih.gov/clinvar/) [33], The Human Gene Mutation Database (http://www.hgmd.cf.ac.uk/ac/index.php) [34], Leiden Open Variation Databases (LOVD) (http://www.lovd.nl/3.0/home) [37], and the Database for Annotation, Visualization and Integrated Discovery (DAVID) (https://david.ncifcrf.gov/) [38] were implemented in the fully detailed bioinformatics pipeline (Fig. 2a).

As results, the total aligned reads was 14,356,459 (father), 17,975,032 (mother) and 20,225,887 (patient). The percentage of reads passing filter that aligned to the reference was 99.9% for all samples. The percentage of targets with coverage greater than 20X was 27.3% for the father, 30.3% for the mother, and 30.4% for the patient with CIPA. Additionally, the analysis of 4811 genes and 18,933 variants was performed in the BaseSpace Variant Interpreter software (Illumina).

After filtering the genomic variants through four steps fully detailed in Fig. 2b, the patient showed a total of 69 pathogenic and/or likely pathogenic variants (Table S3), and 76 variant of uncertain significance (VUS) in 106 genes. The consequence of these 145 variants is fully detailed in Table S4. As results after the DNA genomic analysis, the patient showed a clearly autosomal recessive inheritance pattern with the pathogenic mutation rs763758904 (Arg602*). The other 69 pathogenic and/or likely pathogenic variants in 46 genes were analyzed to better understand the phenotypic heterogeneity of CIPA.

**Fig. 3:**
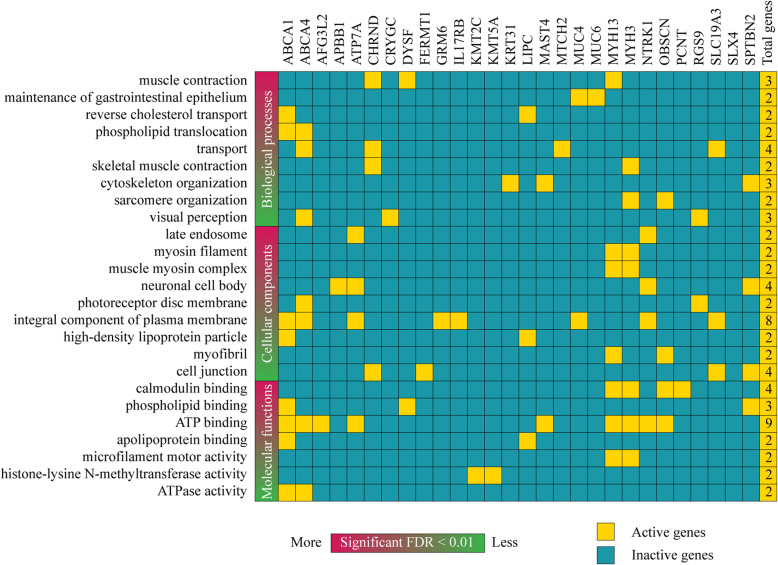
Heatmap of active and inactive genes with pathogenic and/or likely pathogenic variants involved in biological processes, cellular components and molecular functions according to the GO enrichment analysis

Consequently, the enrichment analysis of GO terms related to biological processes, cellular components and molecular functions was carried on in 46 genes with pathogenic or likely pathogenic variants using DAVID Bioinformatics Resource in order to better understand the phenotypic heterogeneity of the patient [38]. Only 28 of 46 genes were involved in almost one of the categories showed as a heatmap in Fig. 3. The most significant biological processes (BPs) with a Benjamini-Hochberg false discovery rate (FDR) < 0.01 were muscle contraction, maintenance of gastrointestinal epithelium, reverse cholesterol transport, phospholipid translocation, skeletal muscle contraction, cytoskeleton organization, sarcomere organization and visual perception. The most significant cellular components (CCs) with a FDR < 0.01 were late endosome, myosin filament, muscle myosin complex, neuronal cell body, photoreceptor disc membrane, integral component o plasma membrane, high-density lipoprotein particle, myofibril and cell junction. In addition, the most significant molecular functions (MFs) with a FDR < 0.01 were calmodium binding, phospholipid binding, ATP binding, apolipoprotein binding, microfilament motor activity, histone-lysine N-methyltransferase activity and ATPase activity [38] (Fig. 3). The function of all these genes is detailed in the Table S5.

**Fig. 4:**
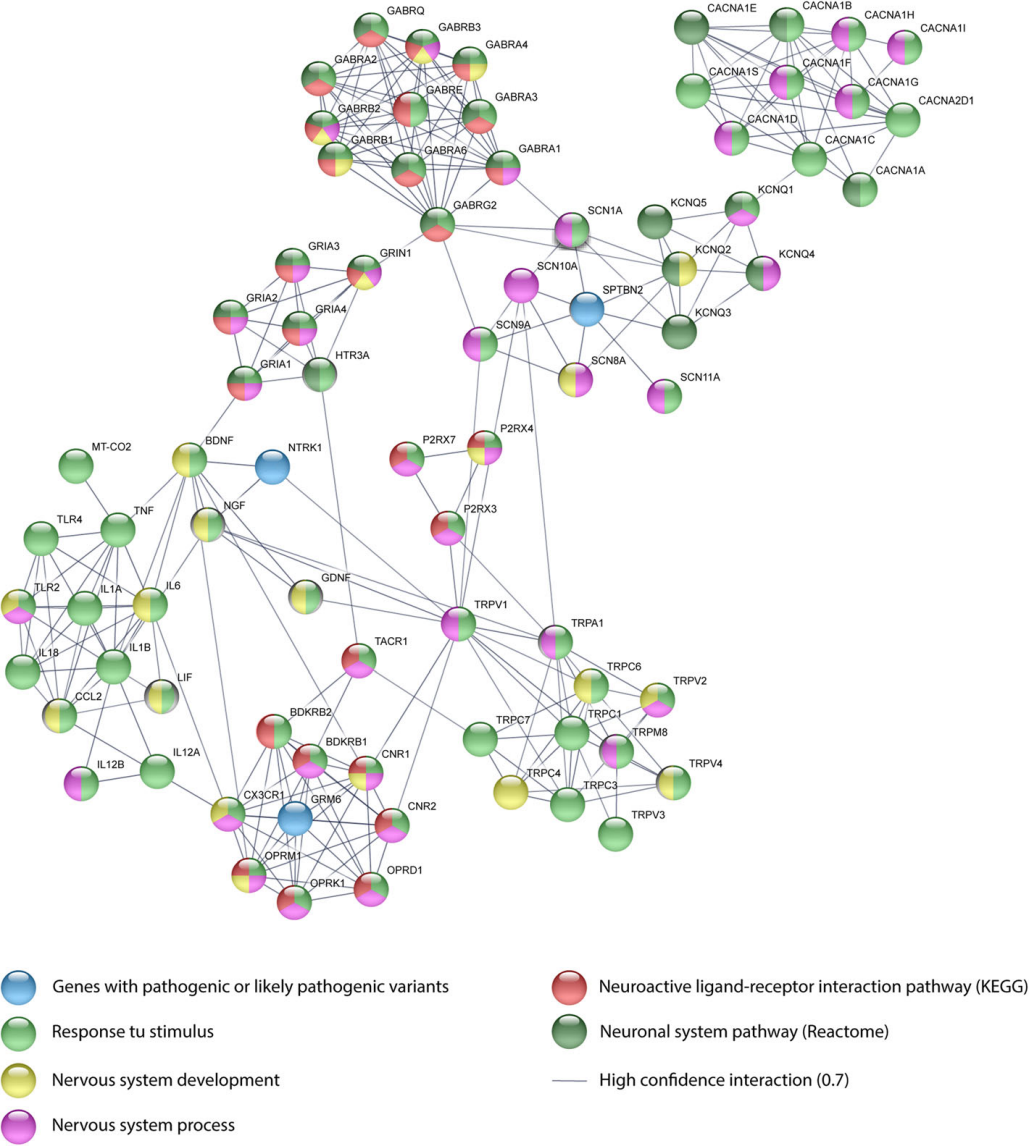
Protein-protein interaction network between the pain matrix genes and genes with pathogenic or likely pathogenic variants

Additionally, a PPi network with a high confidence of 0.7 (*p* < 0.001) was created using String Database [39, 40]. This network was made up of known and predicted interactions between proteins with pathogenic and/or likely pathogenic variants and the pain matrix proteins proposed by Foulkes et al (2008) [10], and detailed in Figure S3 and Table S6. As results, three proteins (NTRK1, GRM6, and SPTBN2) with pathogenic and/or likely pathogenic variants interact with several proteins of the pain matrix. NTRK1 interacts with NGF (peripheral modulation), BDNF (microglia modulation) and TRPV1 (heat transduction). GRM6 interacts with CNR1, OPRD1, OPRK1, OPRM1 (central modulation), CNR2 (peripheral modulation), BDKRB2, BDKRB1 (damage transduction) and CX3CR1 (microglia modulation). Lastly, SPTBN2 interacts with KCNQ3, KCNQ2 (conduction by K^+^ channels), SCN1A, SCN11A, SCN10A, SCN8A and SCN9A (conduction by Na^+^ channels) (Fig. 4).

## Discussion and conclusions

In our study, we reported the first case of a Native American patient from a family who lives in a high-altitude indigenous community (2418 MASL) located north of the Ecuadorian highlands.

Nine years of clinical record have shown that the patient with CIPA has presented several health problems such as bone fractures, self-mutilation, osteochondroma, intellectual disability, Riga-Fede disease, ulcers and fever. One of the strengths of this case was the correct follow-up and adequate intervention and treatment of these health problems, with a positive response and patient tolerance. On the contrary, the biggest limitation of this case is that this autosomal recessive disease has no cure. Regarding patient perspective, parents should share their perspective on the treatments they received.

The *NTRK1* pathway is essential for the maintenance of autonomic sympathetic postganglionic neurons because is responsible for innervating skin through sensory axons [1]. In addition, this pathway is involved in regulate vasoconstriction, sweating, endocytosis and vesicular transport in order to promote neural differentiation [41]. The NTRK1 protein receptor is composed of the extracellular, intracellular, tyrosine kinase domains and a carboxyl terminal tail. NGF ligands bind to NTRK1 receptor stimulating autophosphorylation of tyrosine residues and triggering downstream cell signaling [42]. However, *NTRK1* mutations lead unfavorable survival of pain receptors and sympathetic ganglion neurons [43]. After carrying out the mutational analysis of *NTRK1* and the genomic DNA analysis, the Ecuadorian indigenous patient presented a clearly autosomal recessive inheritance pattern with the pathogenic mutation rs763758904 (Arg602*) and the second missense mutation rs80356677 (Asp674Tyr). rs763758904 (c.1804 C > T; Arg602*) is a pathogenic stop gained / splice region variant characterized by the change of Arg to stop codon. Parents presented the heterozygous genotype (C/T), while the patient presented the homozygous mutant genotype (T/T). This genetic variant has been reported by Wang et al (2016) in a study on five Chinese children with CIPA [44]. On the other hand, rs80356677 (c.2020 G > T; Asp674Tyr) is a missense variant characterized by the change of Asp to Tyr in the amino acid 674. Parents presented the heterozygous genotype (G/T), while the patient presented the homozygous mutant genotype (T/T). This genetic variant has been reported by Indo et al (2001) [19].

The DNA genomic analysis through NGS showed that the CIPA patient presented 69 pathogenic and/or likely pathogenic variants in 46 genes (Table S3). One of these interesting variants was rs324420 in the *FAAH* gene that was identified in a Caucasian female patient with high anandamine concentrations and pain insensitivity [30].

The gene ontology enrichment analysis [38] let us know the possible implication of these 46 genes with the CIPA phenotypic heterogeneity. Only 28 genes were involved in almost one of the categories showed as a heatmap in Fig. 3. The most significant BP was muscle contraction, and transport was the BP with more active genes. The most significant CC was late endosome, and integral component of plasma membrane was the CC with more active genes. The most significant MF was calmodium binding, and ATP binding was the MF with more active genes. Lastly, the GO terms where *NTRK1* was active were late endosome, neural cell body, integral component of plasma membrane and ATP binding.

In regard to the networking analysis showed in Fig. 4, the PPi between proteins with pathogenic and/or likely pathogenic variants and the pain matrix proteins demonstrates that NTRK1 interacts with NGF, BDNF and TRPV1. NGF and BDNF are involved in nervous system development and response to stimulus. TRPV1 is involved in nervous system process and response to stimulus. GRM6 interacts with CNR1, DPRD1, CNR2, BDKRB2, OPRK1, BDKRB1, OPRM1 and CX3CR1. OPRK1, OPRD1, CNR2 and BDKRB1 are involved in nervous system process, response to stimulus and neuroactive ligand-receptor interaction pathway. OPRM1 and CNR1 are involved in nervous system process, response to stimulus, neuroactive ligand-receptor interaction pathway and nervous system development. BDKRB2 is involved in neuroactive ligand-receptor interaction pathway and response to stimulus. CX3CR1 is involved in nervous system process, response to stimulus and nervous system development. SPTBN2 interacts with SCN1A, KCNQ3, KCNQ2, SCN11A, SCN10A, SCN8A and SCN9A. SCN11A, SCN1A and SCN9A are involved in nervous system process and response to stimulus. SCN8A is involved in nervous system development and process. SCN10A is involved in nervous system process. KCNQ2 is involved in nervous system development and the neuronal system pathway. Lastly, KCNQ3 is involved in the neural system pathway [38, 45, 46].

The gene ontology enrichment analysis and the PPi network can contribute to understand how different genes/proteins with pathogenic variants influence the development of phenotypic patterns, symptoms and complications of CIPA patients worldwide.

In conclusion, we conducted for the first time clinical, genomics, PPi networking and GO enrichment analyses in a high-altitude Native American (indigenous) Ecuadorian patient with CIPA and with family history of consanguinity, whose results were associated with the pain matrix in order to find new proteins related to CIPA pathogenesis and phenotypic heterogeneity.

## Supplementary information

**Additional file 1: Figure S1.** Karyotype of patient.

**Additional file 2: Figure S2.** Analysis of *NTRK1* polymorphisms. a) PCR protocol. b) Sanger sequencing analysis protocol.

**Additional file 3: Figure S3.** The pain matrix proteins. We obtained appropriate copyright permission from the corresponding author of the paper (Foulkes et al [10]) to re-design the pain matrix proteins and adapt it as this figure.

**Additional file 4: Table S1.** Studied mutations of the *NTRK1* gene, and their PCR conditions. **Table S2.** TSO full gene list. **Table S3.** Pathogenic and likely pathogenic variants in the CIPA patient after DNA genomic analysis. **Table S4.** Pathogenic variants, probably pathogenic variants and VUS in the CIPA patient after DNA genomic analysis. **Table S5.** Function of genes with at least one pathogenic or likely pathogenic variant on CIPA patient. **Table S6.** Full list of the pain matrix genes.

## Data Availability

All data generated during this study are included in this published article including its supplementary tables. DNA sequences are available in NCBI Sequence Read Archive (SRA) with the BioProject accession number PRJNA647341 (https://www.ncbi.nlm.nih.gov/sra/PRJNA647341). Additionally, database used in this study were Sorting Intolerant From Tolerant (SIFT) (http://sift.bii.a-star.edu.sg/), ClinVar (https://www.ncbi.nlm.nih.gov/clinvar/), The Human Gene Mutation Database (http://www.hgmd.cf.ac.uk/ac/index.php), the Database for Annotation, Visualization and Integrated Discovery (DAVID) (https://david.ncifcrf.gov/), Leiden Open Variation Databases (LOVD) (http://www.lovd.nl/3.0/home), Polymorphism Phenotyping v2 (PolyPhen-2) (http://genetics.bwh.harvard.edu/pph2/), and human genome reference GRCh37 hg19 (https://www.ncbi.nlm.nih.gov/assembly/GCF_000001405.13/).

## Abbreviations

CIPA: Congenital insensitivity to pain with anhidrosis
*NTRK1*: The neurotrophic tyrosine kinase receptor type 1 gene
NGF: Nerve growth factor
SNP: Single nucleotide polymorphism
PPi: Protein-protein interaction
GO: Gene ontology
MASL: Meters above sea level
dNTP: deoxynucleotide triphosphate
FW: Forward
RV: Reverse
TSO: TruSight One
NGS: Next-generation sequencing
VUS: Variant of uncertain significance
LOVD: Leiden Open Variation Databases
DAVID: The database for annotation, visualization and integrated discovery
BP: Biological process
FDR: False discovery rate
CC: Cellular component
MF: Molecular function
