# Supplementary figures and images for: Clinical, genomics and networking analyses of a high-altitude native American Ecuadorian patient with congenital insensitivity to pain with anhidrosis: a case report
Source: BMC Med Genomics. 2020 Aug 17;13:113. doi: 10.1186/s12920-020-00764-3 (PMC7437939; doi:10.1186/s12920-020-00764-3)

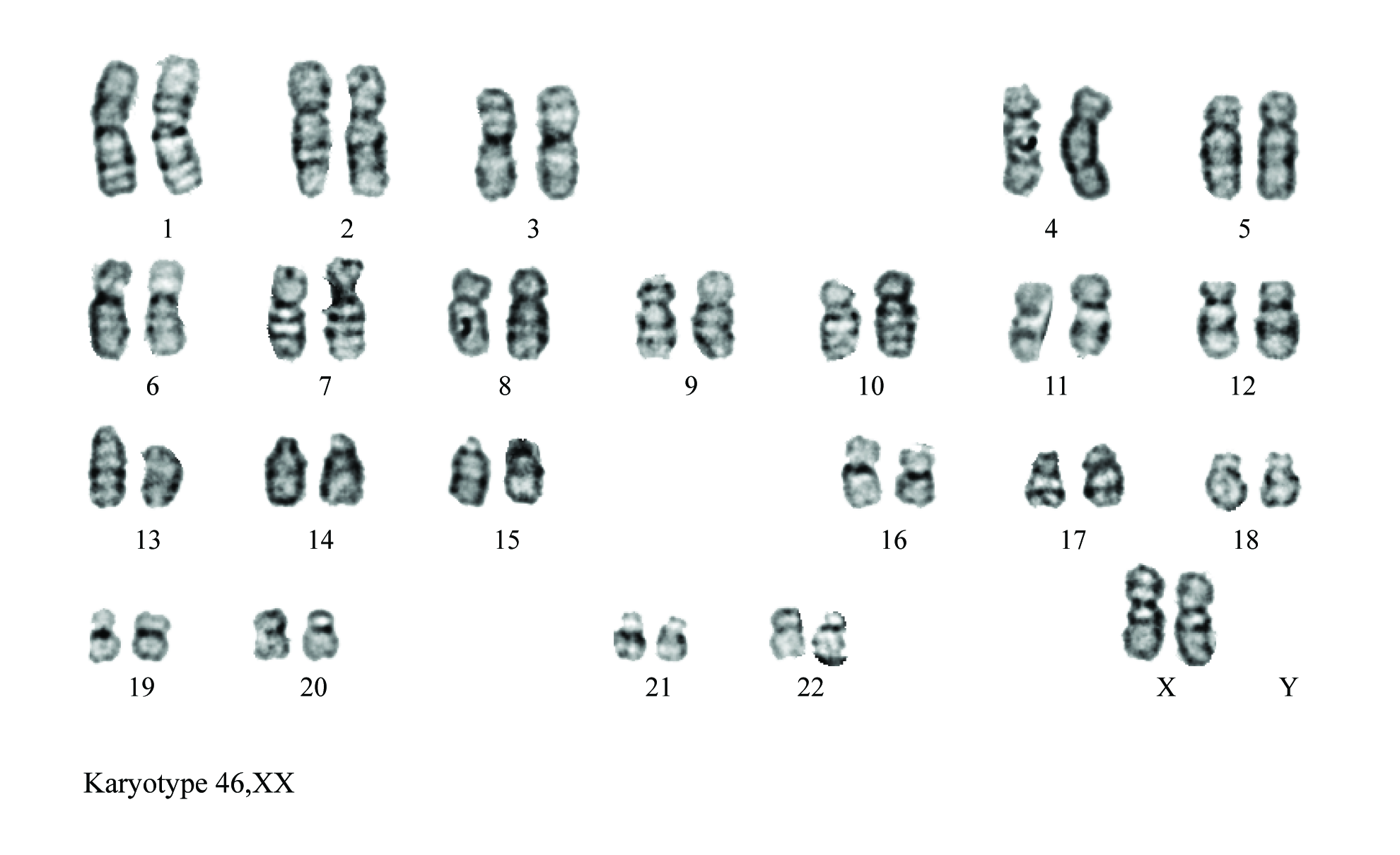

Supplement: Supplementary file 1 — Additional file 1: Figure S1. Karyotype of patient. [file 12920_2020_764_MOESM1_ESM.tif]

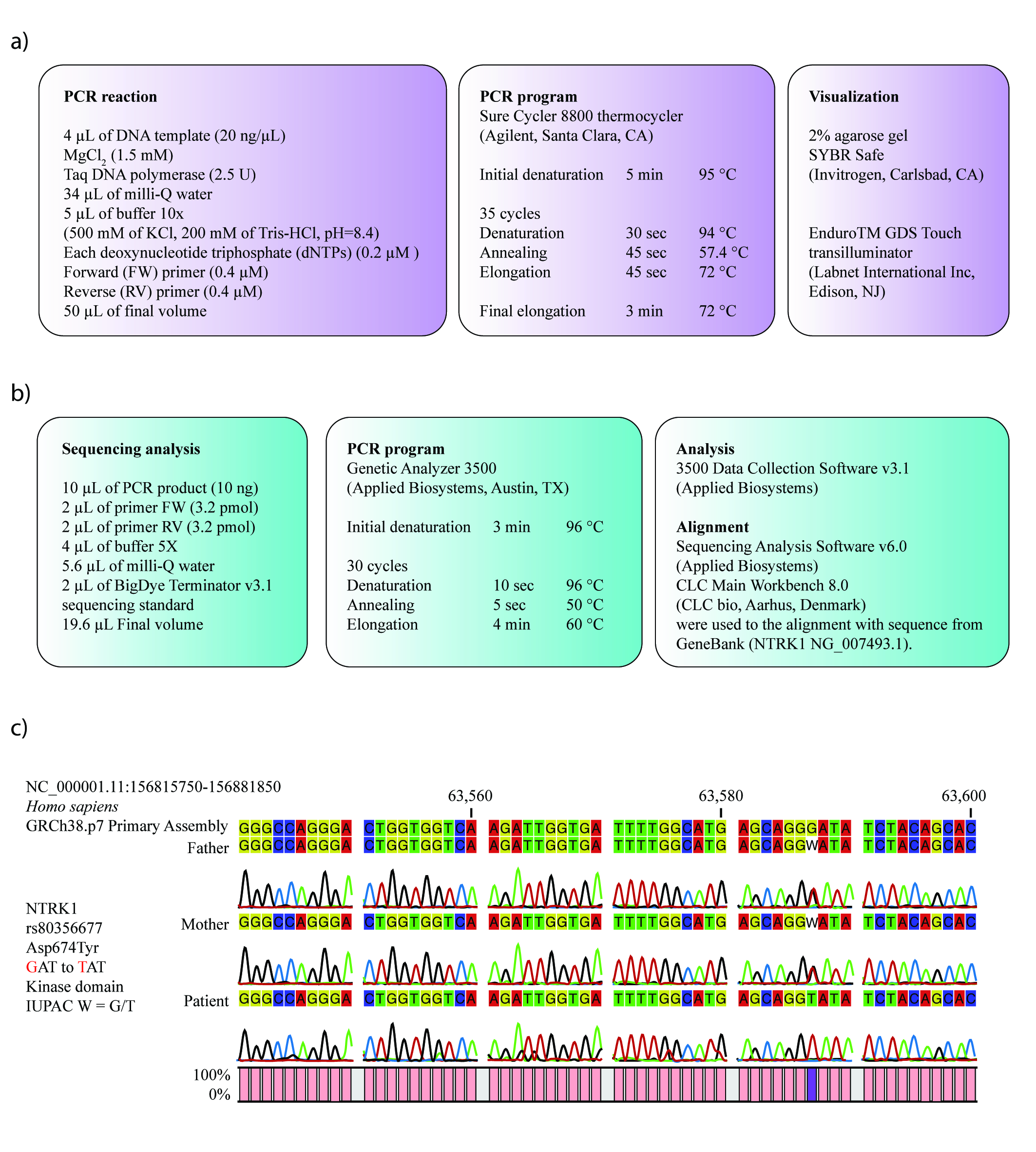

Supplement: Supplementary file 2 — Additional file 2: Figure S2. Analysis of NTRK1 polymorphisms. a) PCR protocol. b) Sanger sequencing analysis protocol. [file 12920_2020_764_MOESM2_ESM.tif]

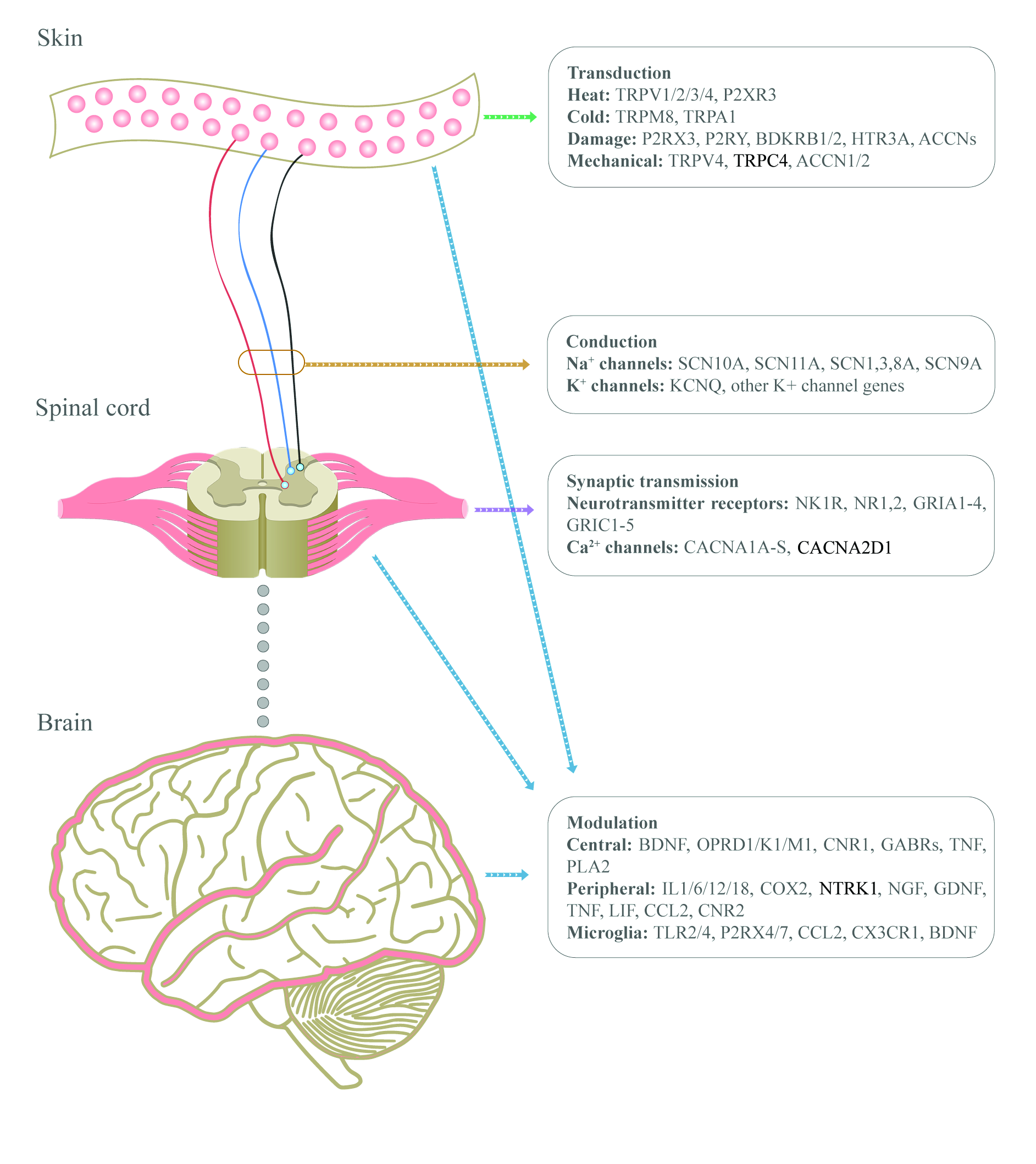

Supplement: Supplementary file 3 — Additional file 3: Figure S3. The pain matrix proteins. We obtained appropriate copyright permission from the corresponding author of the paper (Foulkes et al [10]) to re-design the pain matrix proteins and adapt it as this figure. [file 12920_2020_764_MOESM3_ESM.tif]
